# Future leaders in a learning health system: Exploring the Health System Impact Fellowship

**DOI:** 10.1177/08404704231216951

**Published:** 2023-11-28

**Authors:** Samuel Petrie, Ivy Cheng, Meghan McMahon, John N. Lavis

**Affiliations:** 1 7989 University Health Network, Toronto, Ontario, Canada.; 2 7938 University of Toronto, Toronto, Ontario, Canada.; 3 71545 Sunnybrook Health Sciences Centre, Toronto, Ontario, Canada.; 4 7938 University of Toronto, Toronto, Ontario, Canada.; 5 CIHR Institute of Health Services and Policy Research, Ottawa, Ontario, Canada.; 6 3710 McMaster University, Hamilton, Ontario, Canada.; 7 McMaster Health Forum, Hamilton, Ontario, Canada.

## Abstract

The Canadian health system is reeling following the COVID-19 pandemic. Strains have become growing cracks, with long emergency department wait times, shortage of human health resources, and growing dissatisfaction from both clinicians and patients. To address long-needed health system reform in Canada, a modernization of training is required for the next generation health leaders. The Canadian Institutes of Health Research Health System Impact Fellowship (HSIF) is an example of a well-funded and connected training program which prioritizes embedded research and embedding technically trained scholars with health system partners. The program has been successful in the scope and impact of its training outcomes as well as providing health system partners with a pool of connected and capable scholars. Looking forward, integrating aspects of evidence synthesis from both domestic and international sources and adapting a general contractor approach to implementation within the HSIF could help catalyze learning health system reform in Canada.

## Introduction

According to the pre-pandemic Commonwealth surveys comparing Canada to ten other countries, Canada was a high spender but low performer for access, equity, and health outcomes^1,2^ with increasing and likely unsustainable healthcare costs.^3^ In March of 2022, 2 years into the COVID-19 pandemic, the Canadian Medical Association was of the opinion that Canada’s healthcare system was “collapsing and on life support.”^4^ A year after this announcement, the situation has not improved. There are record highs in staffing shortages,^4^ healthcare worker burnout,^5^ emergency department closures,^6^ and reports of preventable death^7^ as well as increasing health inequities.^8^ The Canadian healthcare system falls far short of achieving the equity-centred quadruple aim.^9^ Additionally, the levels of funding allocated to Canadian health services and policy research as a proportion of total health research funding were low.^10^

In Canada and other jurisdictions, there is increasing attention to the Learning Health System (LHS) and embedded research for evidence-informed learning and improvement. A LHS is one that harnesses data and evidence to respond dynamically to evolving and complex issues.^11^ Embedded research occurs directly within health system organizations (as opposed to primarily in academic institutions), is co-produced between researchers and health decision-makers (e.g., managers, providers, and patients), and is aligned to the operational and strategic needs of the organization to help inform better care and system performance.^12,13^

To strengthen Canada’s embedded research capacity and contribute to advancing towards a LHS, the Institute of Health of Services of Policy Research of the Canadian Institute of Health Services Research (CIHR-IHSPR) collaborated with the Canadian Health Services and Policy Research Alliance to develop the country’s first training modernization strategy for health services and policy research,^14^ which prioritized the importance of building capacity for evidence-informed health system impact. CIHR-IHSPR developed the Health System Impact Fellowship (HSIF) program to address these goals. Our objective is to describe the Health System Impact Fellowship program, its emerging impacts, and potential future directions for evidence-informed health system leadership.^15^

## Genesis and evolution of the Health System Impact Fellowship

The Canadian Institute of Health Services Research developed the HSIF to advance three key objectives: (1) support emerging research leaders to develop their embedded research experience, professional competencies, and potential for impact; (2) grow a strong cadre of embedded researchers positioned to play a key role in evidence-informed health system improvement that advances the Quadruple Aim and health equity; and (3) develop embedded research capacity within health system organizations and catalyze collaborations between academic and health system organizations to contribute to advancing learning health systems across Canada.^10^ Since its inauguration in 2017, 281 Fellows have been embedded in 126 health system organizations and connected to 25 university training programs across Canada.^16^

Applicants to the HSIF are doctoral or post-doctoral students who are embedded into a host health system organization for a 1-year (doctoral level) or 2-year (post-doctoral-level) Fellowship with the goal of advancing the organization’s impact goal with evidence. That is, the program aligns research capacity with the evidence needs of the health system organization. The Fellow has a supervisor who is in a leadership position within the host organization and another from an academic institution who work together to supervise and mentor the Fellow for success and career preparedness.^15^ By being an embedded researcher, the Fellow gains the practical skills of working within the organization and adapting their research skills to address complex real-world problems, and the organization experiences the value of having an embedded researcher to advance achievement of its strategic goals with evidence.^13^

To complement and enrich the Fellows’ experience and impact as an embedded researcher, they receive funds for professional development training to support their development of Canada’s enriched core competencies in HSPR.^16^ The enriched core competency framework features a suite of 10 research and professional competencies that were identified by health system and academic leaders in Canada as essential for emerging research leaders. These competencies facilitate impact and meaningful contributions to evidence-informed health system change.

Fellows select a minimum of three enriched core competencies to develop over the course of the Fellowship based on their individual career goals, which facilitates the creation of a professional development and mentorship plan. The competencies are selected in consultation with their mentors, and both Fellows and mentors meet regularly to reflect on their progress and areas for further attention and growth. Evidence suggests that although Fellows focus on three competencies for development, the Fellowship model (e.g., experiential learning and co-mentorship from a health system and academic leader) provides them with the opportunity to develop a range of skills, with significant improvement observed in all 10 competency areas.^17^ The core competency framework that underpins the HSIF is being refreshed to include skills that the COVID-19 pandemic revealed as critically important, such as, but not limited to equity, scientific communication, and public and patient engagement.^15^

The HSIF program’s downstream impacts are emerging, and program evaluation evidence indicates that impacts include career preparedness for the Fellow in a diverse range of sectors and roles. Further, the program has contributed to a pan-Canadian network of academic and system leaders with a shared commitment to embedded research and the LHS, knowledge creation, increased organizational embedded research capacity, evidence-informed decision-making in the HSIFs partnering health system organizations, and a stronger connection between academia and health system organizations.^15,18^ Evidence on the career pathways of Fellow alum is promising, including research-related leadership roles in academic and system settings.^19^ Additionally, the program has sparked the spread of embedded research models, including Nova Scotia’s Network of Scholars^20^ and the Ontario Health Team (OHT) Impact Fellows program.^21^

## HSIF core competencies: It’s easy to talk the talk but harder to walk the walk

The HSIF offers an example of a training program^17,22^ that allows for trainees to identify and develop important leadership skills crucial to a LHS. Although creating LHS leaders is not an explicit objective of the HSIF, the program could be interpreted as contributing to building foundational skillsets of LHS leaders. The program’s enriched core competency framework can be viewed as a method for building skills across three levels of the health system: micro-, meso-, and macro-level settings. The program could be thought of as providing opportunity to Fellows for exposure to settings and skill development at the micro-level (their individual development within their organization), meso-level (their contribution to their region/provincial contexts) and macro-level (their engagement in a pan-Canadian cohort, including policy leaders and policy issues that are macro in scope). The expansion of the HSIF in 2022 to include international health system organizations (e.g., the World Health Organization and the OECD) provides new opportunity at the macro-level for exposure to global health system challenges, evidence, and leadership.

**Figure 1.**
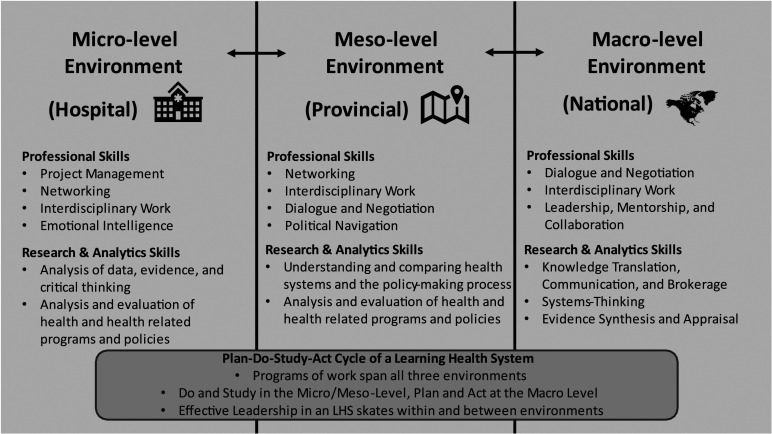
The micro-, meso-, and macro-levels of the health system. A capable leader in a LHS possesses skills which span all three levels. The different levels align with the PDSA cycles foundational to a LHS. The HSIF core competency framework offers a blueprint for fostering and building on important professional and research and analytic skill sets.

Figure 1 provides a representation of Canada’s enriched core competencies and their application across the three levels of a health system. One of the foundational components of a LHS is the focus on quality improvement of services and methods which reflect the dynamic environment in which healthcare is provided.^23^ The identified core competencies will continue to be revised, especially considering the lessons learned from the COVID-19 pandemic and the need to rapidly collate and disseminate evidence to inform advisory and decision-making processes.

### Micro-level environments (institutions, hospitals, and organizations)

Professional competencies at the micro-level include project management, networking, and an ability to work interdisciplinarily. From a research and analytics perspective, analysis of many forms of existing evidence and critical thinking as well as evaluation of health and health-related programs and other contributions to the flow of new evidence are broadly important within day-to-day health system workflows. More generally, a high level of emotional intelligence^24^ is required for impactful work within micro-level environments. While this may seem self-evident to health leaders, what is novel is the articulation of these skills as core competencies. In addition to these professional and analytic core competencies, system thinking abilities are also important to foster.

### Meso-level environments (provincial health authorities and organizations)

The meso-level environment includes the regional or provincial contexts in which a Fellow is situated. Professionally, when comparing the meso to the micro-environment, Fellows need to be effective at dialogue and negotiation in addition to the skills identified in the micro-level environment. The research and analytic skills remain constant, but the audience evaluating the research outputs change. Due to the complex adaptive nature of the health system, the whole of the system is greater than the sum of its parts.^25^ Recognizing that and integrating many forms of evidence in providing evidence support aligned to advisory and decision-making processes within this context, is crucial for the effective governance of a LHS. Further systems thinking capability at the meso-level environment will aid the Fellow (or LHS leader) in recognizing the leverage points that can be used to innovate within the health system.

### Macro-level environments (national health authorities and organizations)

The macro-level environment includes the Fellow’s wider policy, legislative and/or regulatory context, which could feature federal or pan-Canadian, or even international interaction and collaboration. This level of collaboration is difficult to interface with, while paradoxically having the most wide-reaching impact. Professionally, a Fellow's skills within this level should focus on dialogue and negotiation, and interdisciplinary work, as well as leadership in providing evidence for advisory and decision-making processes. Through prior work in the micro and meso-environments, Fellows should recognize the importance of organizational culture and how to navigate it through their micro and meso-level interactions. There is a fundamental shift in the research and analytic skills required when transitioning from the meso-level to the macro-level. Within the macro-level environment, Fellows need to be effective in their evidence support roles, as well as understanding systems thinking methodology and terminology. Further, Fellows should be competent in synthesizing and appraising evidence, ensuring that the correct approaches are being applied in appropriate contexts.

Taken altogether, the core competency framework offers an articulation of important professional, research and analytic skills required to thrive in a LHS. An effective leader can capably navigate the micro, meso, and macro-level environments of a health system and synthesize many forms of domestic evidence, key insights from appropriate global evidence, and select other types of information (jurisdictional scans, lived experiences) to inform best practice within their setting and context.

## Future considerations for a LHS: Integration of general contractors

Effective evaluation^26^, and implementation, within complex environments requires embedded researchers to draw on a range of skills and networks—akin to the “general contractor” within a construction project. That is, to be the convener of the right forms of evidence (e.g., data analytics, behavioural research and implementation research, and qualitative insights) at the right time to advance health system improvement and impact for the “homeowner” (policy-maker).^26^

To illustrate the embedded researcher as a “general contractor” metaphor, consider a pilot project seeking to implement a virtual care intervention to remotely monitor congestive heart failure patients in rural and remote Indigenous communities. For there to be successful implementation of the intervention, the project requires meaningful and culturally safe engagement with Indigenous Peoples and communities, effective collaboration between many different divisions (or silos) within the health system, such as cardiology, digital health, rural and remote practice, and primary care, among others, and adherence to Ownership, Control, Access, and Possession OCAP® principles of Indigenous data sovereignty. The people involved in the project may prioritize different forms of domestic evidence (e.g., modelling and evaluation), global evidence (i.e., what have we learned from around the world, including how it varies by groups and contexts), and other types of evidence like lived experience and Indigenous ways of knowing.

The multi-faceted components of this project are not unique to the example. Other interventions and implementations may vary in stakeholders, timelines, and outcomes of interest; however, the fact remains that the richness of the health system creates complex and dynamic environments which require robust approaches. Quickly, a relatively small program can become an unmanageable wicked problem.^27^ An embedded researcher with breadth of knowledge in research approaches and a range of skills—including co-design, cross-sectoral collaboration, Indigenous health research, implementation science, change leadership, and an extensive network to draw upon for skills in areas they do not personally hold—is needed. This could be viewed as akin to a general contractor who pulls together a range of skills and expertise to deliver on a concrete goal.

The siloed nature of the health system has been discussed and documented extensively.^28–30^ Increasingly, embedded scientists are being leveraged to provide evidence support aligned to advisory and decision-making processes.^31,32^ These embedded scientists have significant technical expertise and can leverage this to contribute to a breadth of complex health system challenges, integrating evidence for their context from domestic and global evidence sources and from other ways of knowing. Further, they are aware of the dynamic context a program must succeed in, so their recommendations are achievable and realistic for the setting.^33^

## Conclusion and future directions

The Health System Impact Fellowship is one component of the broader health ecosystem and one contributor to the advancement of the LHS. The program has the specific aim of developing embedded researcher expertise and capacity. Although the HSIF program has shown promise in its early outcomes, the program itself must continue to adapt and evolve. Adopting the forthcoming refreshed core competency framework and integrating the updated competencies into the NCTP will help to ensure Fellows receive state-of-the-art training in areas deemed high priority to health system leaders. Incorporating skill development in evidence support strategies, such as rapid evidence synthesis and policy dialogues may help to ensure embedded researchers incorporate the best available global evidence with domestic evidence and local context and share it with their partner organizations in the most appropriate, tailored, useable format.

Incorporating evidence support skills may also help prepare embedded researchers to contribute to advisory and decision-making processes beyond their embedding organization and within macro-level environments of health policy, elevating and expanding the potential reach and impact of the embedded researcher model. Understanding how to explicitly link to advisory and decision-making processes and putting evidence into relevant systems, policy, equity and other frameworks are important aspects of engaging with policy-makers within the macro domain. To conclude, promotion of core research and leadership competencies, system thinking skills, and a general contractor perspective may also contribute to breaking down the siloed aspects of the health system and help catalyze LHS reform.
